# The causal relationship between enlarged perivascular spaces and intracerebral hemorrhage: A 2-sample Mendelian randomization study

**DOI:** 10.1097/MD.0000000000042658

**Published:** 2025-06-06

**Authors:** Wentao Yan, Xiuhua He, Guochao Hu, Kui Chen, Guanjun Wang

**Affiliations:** a Department of Neurosurgery, Xuchang Central Hospital, Xuchang, China; b Department of Cardiovascular Medicine, Xuchang Central Hospital, Xuchang, China.

**Keywords:** basal ganglia, brain, cerebral hemorrhage, glymphatic system, Mendelian randomization analysis

## Abstract

The genetic prediction of the causal relationship between enlarged perivascular spaces (PVS) and intracerebral hemorrhage (ICH). We performed a 2-sample Mendelian randomization (MR) study that used published data from genome-wide association studies on ICH and PVS. We primarily utilized the inverse variance weighted (IVW), MR-Egger, weighted median and weighted mode method. Sensitivity analyses included Cochran *Q* test, MR-Egger regression, MR-PRESSO global test and leave-one-out analysis. IVW analysis showed no statistical association between genetically predicted enlargement of hippocampal PVS (OR = 0.74, 95% CI = 0.23–2.35, *P* = .605), basal ganglia PVS (OR = 1.59, 95% CI = 0.64–3.95, *P* = .318), or white matter PVS (OR = 1.59, 95% CI = 0.64–3.95, *P* = .318) with the risk of ICH. The results of MR-Egger regression, Weighted Median, and Weighted Mode methods were consistent with those of the IVW method. The sensitivity analyses did not reveal any pleiotropy or heterogeneity. The leave-one-out plots did not found any single mutation that might influence the results. Our findings indicate that there is no causal relationship between PVS enlargement and the development of ICH at the genetic level. Using PVS as a diagnostic marker might lack specificity, needed for the planning of timely diagnostic procedures in the risk populations.

## 1. Introduction

Stroke remains a leading cause of morbidity and disability worldwide, primarily classified into ischemic and hemorrhagic subtypes.^[1]^ Of these, intracranial hemorrhage (ICH) is the rarer of the 2 stroke subtypes but presents a particularly lethal form of stroke, with a notably high incidence of 29.9 per 100,000 person-years, which has been steadily increasing worldwide over the last 20 years.^[2,3]^ While ICH accounts for a relatively small percentage of overall stroke cases, it has disproportionately high morbidity and mortality, with 2-year mortality rates reaching up to 49.5%.^[4]^ In addition to the high mortality, only 1/6th of ICH patients are discharged home independently and over 1/3rd are discharged to long-term care facilities, contributing significantly to early disability burden.^[5]^ The ICH is more prevalent in the Asian population, men, and older individuals, particularly those with hypertension; it is also twice as common in low- to upper-middle-income countries compared to high-income countries.^[3]^ Multiple other factors affect the recovery and health-related quality of life of ICH survivors, many of which remain understudied.^[5]^ Thus, despite extensive research on diagnostic and management strategies, the prognosis for ICH remains grim.

Perivascular spaces (PVS) encompass a variety of passageways around arterioles, capillaries, and venules in the brain that play a critical role in maintaining homeostasis, primarily by clearing cerebrospinal fluid (CSF) and waste.^[6]^ When enlarged, PVS become visible, which previous observational and experimental studies have linked to the pathogenesis of small vessel disease, Alzheimer disease, and other neurodegenerative and inflammatory disorders.^[7]^ Additionally, PVS morphology has been shown to influence cognitive function, vascular risk factors, vascular and neurodegenerative brain lesions, sleep patterns, and cerebral hemodynamics.^[6,8]^ In ICH patients, the diagnostic role of PVS remains under discussion. One study reported an association between enlarged PVS and recurrent ICH,^[9]^ while another found a higher risk of vascular death, but not the incidence of stroke, after adjusting for vascular risk factors.^[10]^ Moreover, PVS are largely absent in histological sections, and the use of CSF tracers is significantly limited in postmortem samples, where it inevitably reflects interstitial fluid movements after death.^[8]^ More data are required to determine the clinical relevance of PVS and the associated risks of PVS enlargement in ICH.

Utilization of Mendelian randomization (MR) methods may help to overcome the limitations of *ex vivo* research and reverse causality of epidemiological studies by deriving evidence for a direct causal relationship.^[11]^ One of the important assumptions of MR method is that genome-wide association studies (GWAS) can identify genetic variants that are strongly associated with the biomarker of interest, allowing single nucleotide polymorphisms (SNPs) to serve as instruments indicating lifetime exposure.^[12]^ Previous MR studies of ICH discussed the relationship between the age at menarche with the risk of ICH, reporting the strong association with nonlobar ICH,^[13]^ while the relationship between ischemic colitis and Crohn disease with ICH was debated, despite observational evidence.^[14]^ Application of MR could minimize the risks for patients and experimental costs, especially in cases where the hypothesized association is controversial or debatable.^[11,12]^ However, no MR correlation studies have been performed for ICH and PVS, so whether PVS enlargement can be used as a predictor of ICH is a choice topic worthy of exploration in MR analysis.

Given the uncertainty about the causal association between PVS enlargement and ICH, our study used genetic prediction instruments to investigate the potential causal relationship between enlargement of PVS and the risk of ICH, in order to assess viability of hypothesis that PVS morphology could be used as a potential diagnostic marker in the future studies.

## 2. Materials and methods

### 2.1. Study design

**Figure 1.**
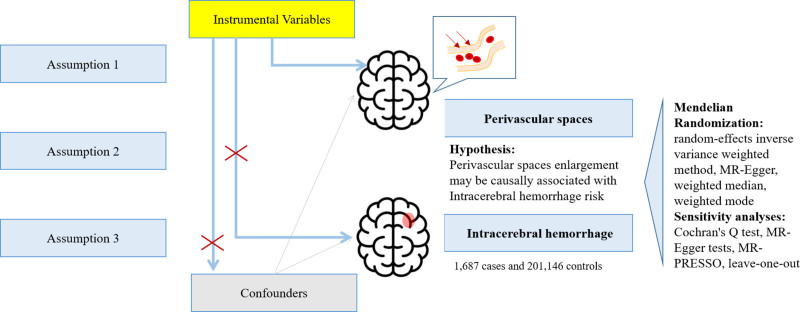
Flow diagram illustrating the analytical process of the 2-sample MR study investigating the causal relationship between Enlarged Perivascular Spaces and Intracerebral Hemorrhage. The figure outlines the selection of genetic instrumental variables from publicly available summary statistics, the analytical workflow of the MR study, and the 3 core assumptions underlying MR: relevance (strong association with exposure), independence (no association with confounders), and exclusion restriction (influence on outcome only through exposure). MR = Mendelian randomization.

In this study, we performed a 2-sample MR analysis to examine the causal effects of PVS enlargement on ICH. SNPs selected as instrumental variables (IVs) for this study were sourced from publicly available GWAS summary data related to exposures and outcomes of interest. MR has to fulfill 3 core assumptions: the genetic IVs should be strongly associated with the exposure; the genetic IVs are not associated with confounders linked to the chosen exposure and outcome; the genetic IVs affect the outcome only via exposure and not directly or through confounding factors (Fig. 1).

This study was conducted according to the Strengthening the Reporting of Observational Studies in Epidemiology (STROBE) reporting guideline.^[15]^ Additional ethical approval was deemed unnecessary for this study due to the summary statistics from published accessible GWAS data.

### 2.2. Data source

The GWAS data for ICH were obtained from the IEU database analysis of the FinnGen large cohort published by Qing et al^[16]^ in 2024, and included 1687 cases and 201,146 controls, all of European ancestry.

The GWAS data for PVS enlargement included SNPs associated with extensive hippocampal perivascular space burden (9163 cases, 29,708 controls), extensive basal ganglia perivascular space burden (8950 cases, 29,953 controls) and extensive white matter perivascular space burden (9317 cases, 29,281 controls), all extracted from the study by Duperron et al^[17]^ published in 2023; based on the previous report that PVS morphology notably differ across racial/ethnic cohorts,^[18]^ only data for European ancestry participants was utilized.

### 2.3. Selection of instrumental variables

To filter eligible genetic IVs that fulfill the 3 core MR assumptions, we performed a set of quality control techniques. SNPs demonstrated a strong association with PVS enlargement and ICH with a significance level of *P* < 5 × 10^−8^. Only SNPs with a minor allele frequency >0.01 were retained.^[19]^ SNP linkage disequilibrium (LD) was addressed by removing SNPs based on an R^2^ threshold of <0.001, using a window size of 10,000 kb.^[19]^ In cases where a selected IV was absent from the summary statistics of the outcome, a proxy SNP was sought with high LD with the original IV, specifically with an R^2^ value >0.8.^[11]^ To evaluate the strength of each SNP serving as an IV and to mitigate potential weak instrument bias, the *F*-statistic was calculated. This measure assesses whether an IV effectively explains the variation in the exposure (use of anesthesia during childbirth). The formula for calculating the *F* value is: *F* = *R*^2^ × (N − 2)/(1 − *R*^2^), where *R*^2^ represents the proportion of variance in the exposure explained by the SNP within the IV. The requirement for the *F* value was to be >10 to ensure that the IV was strong enough to avoid potential biases.^[20]^

### 2.4. MR analysis

This analysis employed the random-effects inverse variance weighted (IVW) method as the primary approach to assess the causal relationship between the exposure and the risk of the outcome, calculating odds ratios and their corresponding 95% confidence intervals.^[19]^ The IVW was also the principal method for interpreting MR results, calculating a weighted average of effect sizes across SNPs, with weights being the inverse of the variance for each SNP. Additionally, robustness checks were performed using MR-Egger, weighted median, and weighted mode methods. The MR-Egger method accounts for the presence of an intercept term and provides accurate causal effect estimates even in the presence of directional pleiotropy.^[21]^ The weighted median method assumes that at least half of the IVs are valid and examines the causal relationship between the exposure and the outcome.^[22]^ All analyses in this study were conducted using the “TwoSampleMR” package of R version 4.3.0. Additionally, this study employed forest plots, scatter plots, and funnel plots for visual analysis of the results. A *P*-value < .05 was deemed statistically significant.

### 2.5. Sensitivity analysis

Sensitivity analyses were employed in this study to detect potential pleiotropy and heterogenity. Specifically, Cochran *Q* test was used to assess heterogeneity among IVs with *P* > .05 indicating low heterogeneity and suggesting that variations in estimates among the IVs were random and had minimal impact on the IVW results. To further address the influence of pleiotropy on the estimated causal effects, the MR-Egger regression method was adopted. If the intercept term in the MR-Egger regression approached zero or was statistically nonsignificant, it suggested no evidence of directional pleiotropy affecting the results.^[21]^ Additionally, the MR-pleiotropy residual sum and outlier (MR-PRESSO) method was implemented to identify and examine potential outliers (i.e., SNPs with *P* < .05) that might indicate horizontal pleiotropy. Upon identifying such outliers, they were removed, and the causal associations were reestimated to correct for the effects of pleiotropy. Lastly, a leave-one-out analysis was conducted to evaluate the robustness and consistency of the findings. This method involves iteratively excluding 1 SNP at a time from the analysis to determine if the overall conclusion remains stable without reliance on any single SNP.^[23]^

## 3. Results

### 3.1. IVs selection

In this study, 41 IVs related to PVS enlargement were screened. The mean value of *F*-statistic of IV was calculated as 34.18, minimum value of *F*-statistic was 14.66 and maximum value was 90.78 (Table S1, Supplemental Digital Content, https://links.lww.com/MD/P62).

When MR analysis was performed with extensive white matter PVS burden as the exposure, 14 IVs were selected with the mean *F*-statistic of 38.94 (21.78, 90.78), and 1 weak IV (rs13079464) was excluded. For extensive basal ganglia PVS burden as the exposure, 14 IVs were selected with the mean *F*-statistic of *F* = 38.94 (21.78, 90.78). For extensive hippocampal perivascular space burden as exposure, 13 were selected with the mean *F*-statistic of 23.93 (14.66, 49.38) and. 2 SNPs (rs11500477, rs2022392) were not matched with information in the summary data. The rs11500477 was replaced with rs10769263 as proxy SNP (*R*^2^ = 0.0005641596) and rs2022392 was replaced with rs20560 as proxy SNP (*R*^2^ = 0.0012687212). One weak IV (rs2022392) was excluded. All SNPs included as IVs in this study are listed in Table S2, Supplemental Digital Content, https://links.lww.com/MD/P64.

### 3.2. MR analysis results

**Figure 2.**
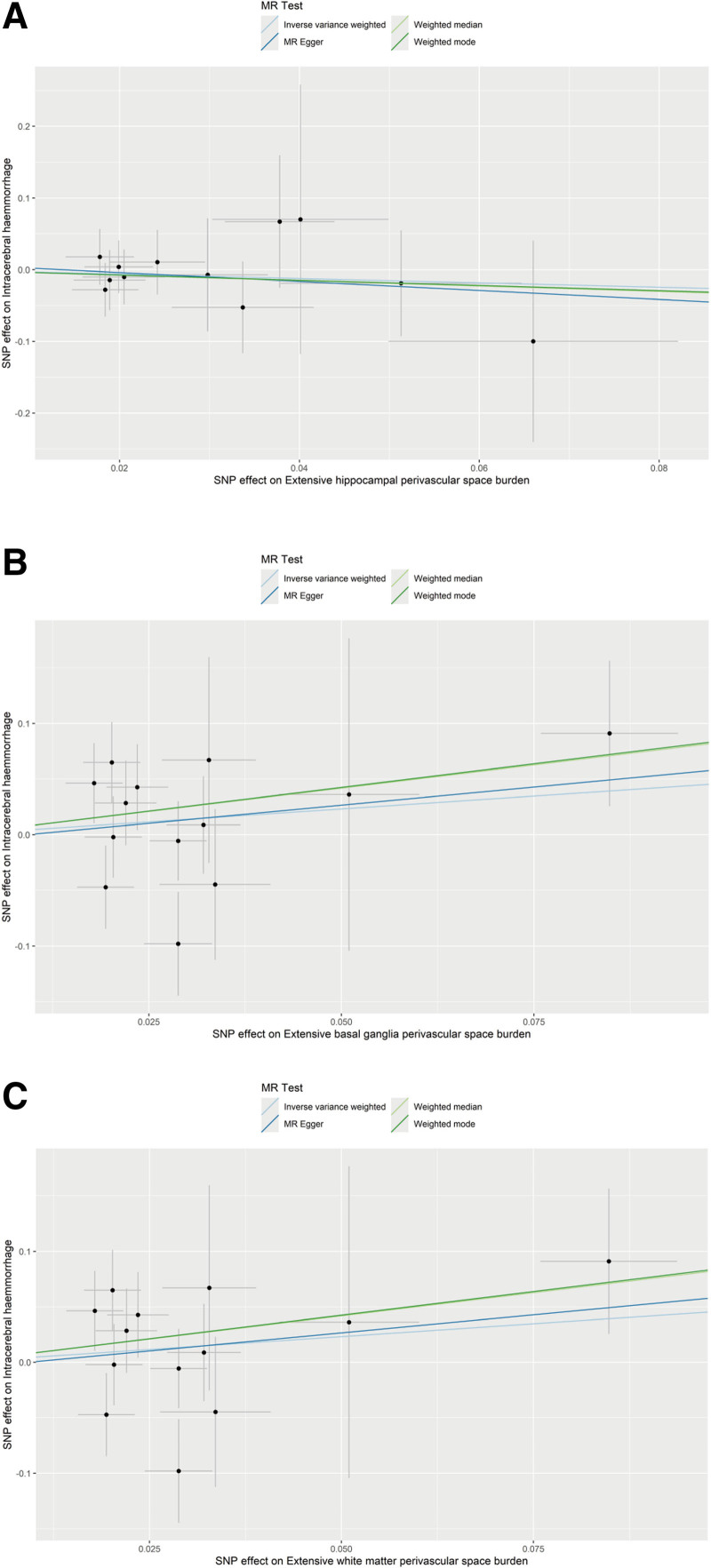
Scatter plots of the association between enlarged perivascular spaces and intracranial hemorrhage in the MR analysis. (A) Extensive hippocampal perivascular space burden, (B) extensive basal ganglia perivascular space burden, (C) extensive white matter perivascular space burden. MR = Mendelian randomization.

**Figure 3.**
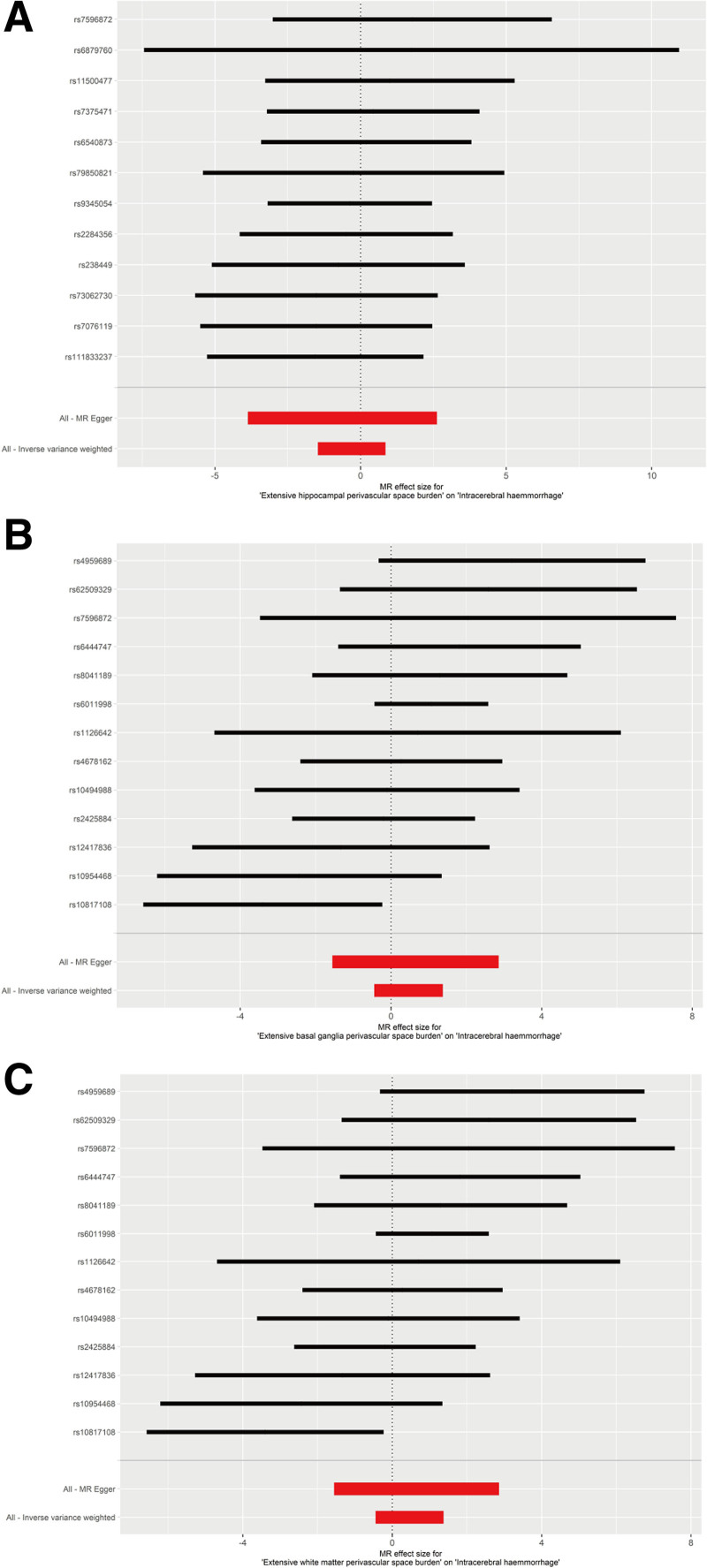
Forest plots of the association between enlarged perivascular spaces and intracranial hemorrhage in the MR analysis. (A) Extensive hippocampal perivascular space burden, (B) extensive basal ganglia perivascular space burden, (C) extensive white matter perivascular space burden. MR = Mendelian randomization.

IVW analysis indicated that no statistically significant association between hippocampal PVS (OR = 0.74, 95% CI = 0.23–2.35, *P* = .605), basal ganglia PVS (OR = 1.59, 95% CI = 0.64–3.95, *P* = .318), or white matter PVS (OR = 1.59, 95% CI = 0.64–3.95, *P* = .318) with the risk of ICH, as shown in Figure 2. The result of weighted median analysis, MR-Egger and weighted mode analysis were consistent with the IVW method (Table 1). Effect sizes for causal associations are demonstrated on Figure 3.

**Table 1:** Results of MR analyses evaluating the potential causal relationship between enlarged perivascular spaces in different brain regions and the risk of intracerebral hemorrhage.

Exposure	Significant of SNP	Number of SNPs	Methods	OR (95% CI)	*P*
Extensive hippocampal perivascular space burden	5 × 10^−8^	12	Inverse variance weighted	0.74 (0.23–2.35)	.605
		12	MR-Egger	0.54 (0.02–13.74)	.714
		12	Weighted median	0.68 (0.16–2.95)	.611
		12	Weighted mode	0.69 (0.09–5.27)	.731
Extensive basal ganglia perivascular space burden	5 × 10^−8^	13	Inverse variance weighted	1.59 (0.64–3.95)	.318
		13	MR-Egger	1.92 (0.21–17.43)	.575
		13	Weighted median	2.31 (0.69–7.70)	.173
		13	Weighted mode	2.34 (0.61–8.94)	.237
Extensive white matter perivascular space burden	5 × 10^−8^	13	Inverse variance weighted	1.59 (0.64–3.95)	.318
		13	MR-Egger	1.92 (0.21–17.43)	.575
		13	Weighted median	2.31 (0.71–7.52)	.165
		13	Weighted mode	2.34 (0.62–8.90)	.235

Estimates are derived using 4 complementary MR methods: IVW, MR-Egger, weighted median, and weighted mode, providing a robust assessment of causal inference.

CI = confidence interval, IVW = inverse variance weighted, MR = Mendelian randomization, OR = odds ratio, SNP = single nucleotide polymorphism.

### 3.3. Sensitivity analysis results

**Figure 4.**
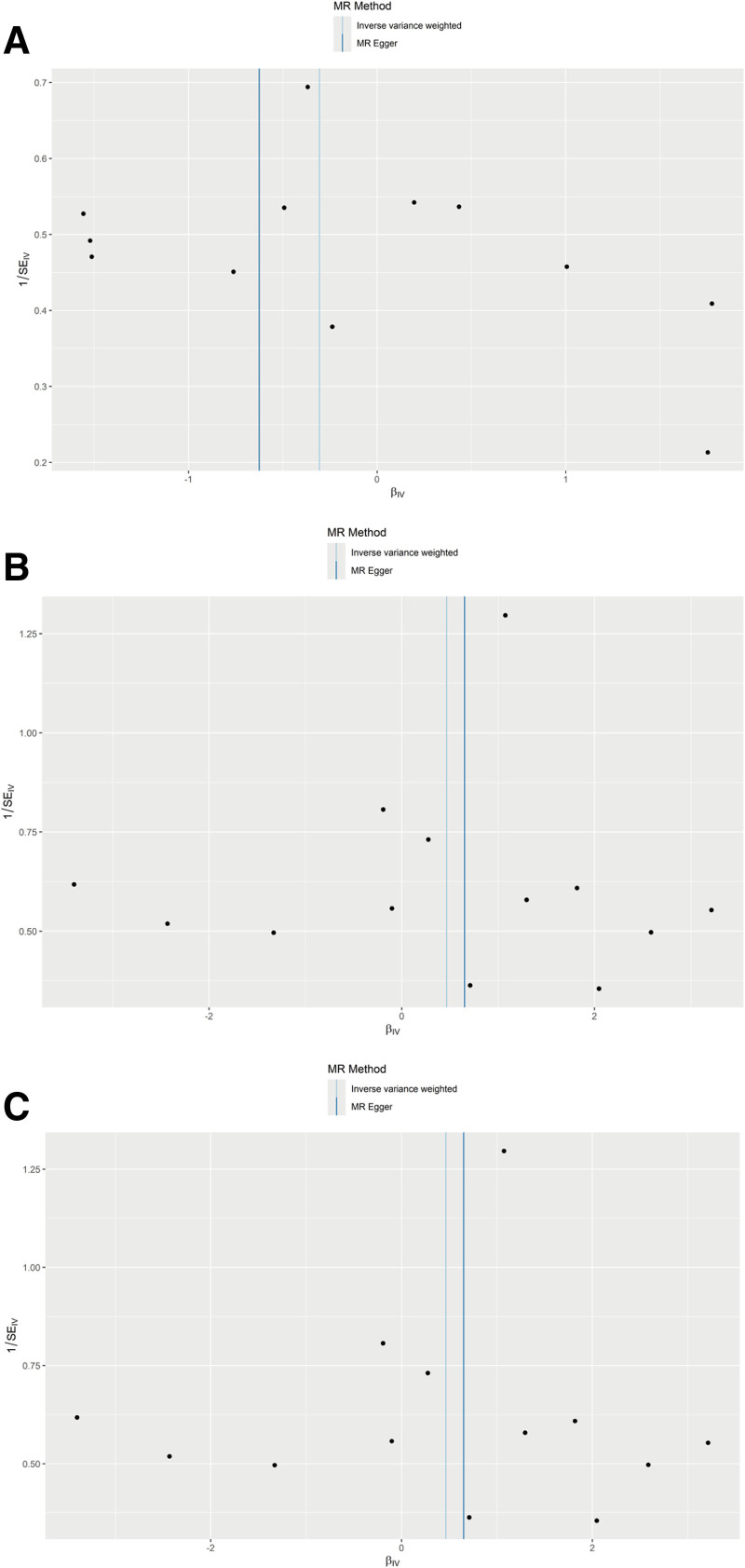
Funnel plots of the association between enlarged perivascular spaces and intracranial hemorrhage in the MR analysis. (A) Extensive hippocampal perivascular space burden, (B) extensive basal ganglia perivascular space burden, (C) extensive white matter perivascular space burden. MR = Mendelian randomization.

**Figure 5.**
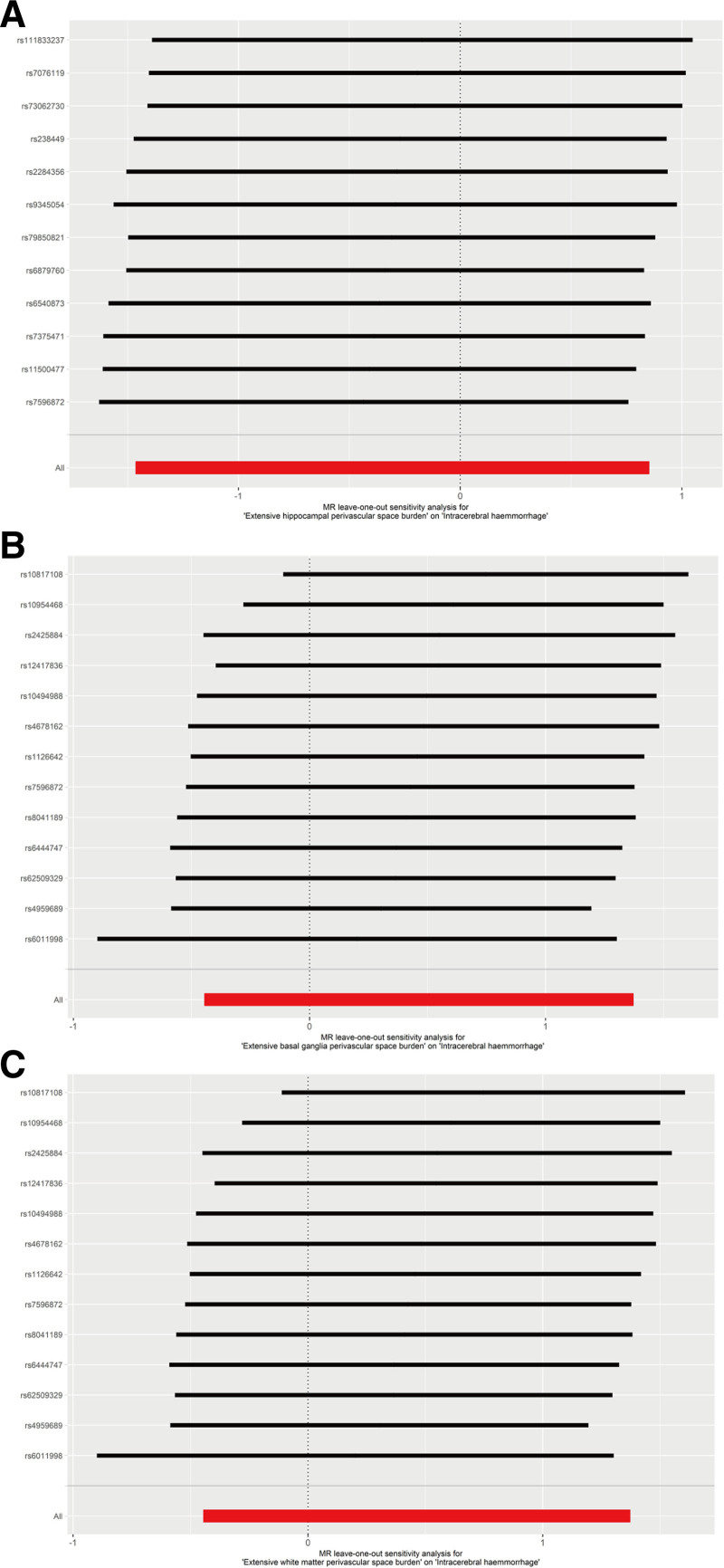
Leave-one-out sensitivity analysis assessing the robustness of MR results for the causal effect of enlarged perivascular spaces on intracerebral hemorrhage. Each plot illustrates the impact of excluding individual single nucleotide polymorphisms (SNPs) on the overall causal estimate: (A) extensive hippocampal perivascular space burden, (B) extensive basal ganglia perivascular space burden, and (C) extensive white matter perivascular space burden, indicating the stability of the associations across all instrumental variables. MR = Mendelian randomization.

Sensitivity analyses showed that heterogeneity was not significant for all exposure and outcome analyses (Table 2) and effect of horizontal pleiotropy was insignificant (Table 3). The funnel plots of the MR-Egger regression also indicated that this analysis was not affected by horizontal pleiotropy (Fig. 4). The leave-one-out plots did not found any single SNPs that might influence the results (Fig. 5).

**Table 2:** Sensitivity analyses assessing the validity of instrumental variables used in MR analyses of enlarged perivascular spaces and intracerebral hemorrhage.

Outcome	Exposure	Heterogeneity			Pleiotropy		
		*Q* statistic (IVW)	*P*-value	FDR adjusted *P*-value	MR-Egger Intercept	*P*-value	FDR adjusted *P*-value
Intracerebral haemmorrhage	Extensive hippocampal perivascular space burden	2.68	.994	.994	0.008	.841	.857
Intracerebral haemmorrhage	Extensive basal ganglia perivascular space burden	14.45	.273	.409	−0.006	.857	.857
Intracerebral haemmorrhage	Extensive white matter perivascular space burden	14.45	.273	.409	−0.006	.857	.857

Results include heterogeneity testing using the IVW *Q* statistic and horizontal pleiotropy assessment via MR-Egger intercept, indicating no significant violations across all exposure-outcome pairs.

CI = confidence interval, FDR = false discovery rate, IVW = inverse variance weighted, MR = Mendelian randomization, OR = odds ratio.

**Table 3:** MR-PRESSO analysis for detection of horizontal pleiotropy and outlier effects in the causal relationship between enlarged perivascular spaces and intracerebral hemorrhage.

Exposure	Outcome	Raw		Outlier corrected		Global *P*	Number of outliers	Distortion *P*
		OR (CI%)	*P*	OR (CI%)	*P*			
Extensive basal ganglia perivascular space burden	Intracerebral haemmorrhage	1.59 (0.64–3.95)	.337	NA	NA	.282	NA	NA
Extensive white matter perivascular space burden	Intracerebral haemmorrhage	1.59 (0.64–3.95)	.337	NA	NA	.283	NA	NA
Extensive hippocampal perivascular space burden	Intracerebral haemmorrhage	0.74 (0.42–1.31)	.317	NA	NA	.992	NA	NA

The results indicate no evidence of outliers or significant distortion effects, supporting the robustness of the MR findings.

CI = confidence interval, MR-PRESSO = Mendelian randomization-pleiotropy residual sum and outlier, OR = odds ratio.

## 4. Discussion

This study applied a 2-sample MR analysis and utilized most recent data on genetic variants associated with the morphology of cerebral PVS, but no conclusive evidence has been found of causal relationship between PVS enlargement and ICH. Although previous observational studies reported that visible/enlarged PVS were strongly associated with the increased risk of stroke, alone with hypertension, systemic inflammation and dementia,^[6,24]^ results of the present study cannot support the causality of this connection. It is most probable that observed association is heavily influenced by confounders that need to be further discussed; nevertheless, findings of this study contribute to the ongoing search for effective ICH management strategies, and could lessen the burden of excessive diagnostic procedures in the risk populations.

Age is one of the most notable confounders influencing both PVS morphology and the development of ICH, which is projected to rise with an aging population.^[25]^ In particular, association between PVS enlargement and ICH has been clinically reported in the study by Duperron et al,^[26]^ where PVS visibility was associated with a higher risk of developing ICH (HR, 3.12 [1.78–5.47]) after adjusting for intracranial volume increase; results seemingly contradicted by present study findings. However, in Duperron study the mean age of participants was 72.7 ± 4.1 years, while another study noted that in healthy individuals PVS enlargement was significantly more common in older age groups.^[27]^ Conversely, MR models lifetime exposure, suggesting the moderate enlargement of PVS is not causally associated with the development of ICH. It is in line with the most recent magnetic resonance study by Best et al^[28]^ where, after adjustment for age, highly visible PVS were associated with higher ischemic stroke risk but not ICH risk. As PVS enlargement was also previously shown to reflect cerebral amyloid angiopathy and atherosclerotic changes not limited to older age,^[27,29]^ it could be hypothesized that patients with vascular risk factors would present enlarged PVS more often, which warrants future research.

Secondly, sleep has been shown to regulate brain waste clearance via the PVS, and may thereby influence PVS morphology as a potential confounding factor.^[18,30]^ Experimental studies have demonstrated that sleep is associated with increased PVS size and enhanced convective flow of CSF in mice, suggesting that sleep pattern could notably change PVS size and shape.^[29]^ Clinical evidence further supports this notion, showing associations between sleep and higher PVS load in the centrum semiovale^[30]^ as well as increased PVS volume fraction,^[18]^ dynamically changing the PVS functionality. In addition, body mass index was found to mediate the effect of sleep quality on PVS morphology in younger individuals but not in the elderly,^[18]^ suggesting that PVS dynamically change under the cumulative influence of age, sleep and metabolic function. This dynamic nature raises questions about the suitability of PVS as a universal static biomarker. This is further supported by recent research highlighting the complex interplay between neuroinflammation, immune response, and neurovascular integrity in neurodegenerative conditions and stroke. For instance, T cells and tyrosine kinase receptors, both implicated in neuroplasticity and neuroimmune modulation, may also contribute to variations in morphology.^[31,32]^ Given the multifactorial and evolving nature of brain microenvironments, including vascular and immune influences, the reliability of PVS as a static biomarker across diverse populations and disease states remains limited.

Finally, PVS are more likely to reflect impaired brain waste clearance due to some diseases and conditions, rather than predate pathological events. It is hypothesized that PVS serve as the highways for clearance of metabolic waste products from the brain, which may represent the activity of the glymphatic system, and was therefore considered as possible clinical neuroimaging biomarker of various neurological disorders.^[7]^ However, the critical sets of data have yet to be collected, leading to the controversy in discussion.^[8]^ Present study used most recent GWAS data on the PVS morphology and findings do not support the decisive role of PVS enlargement in the development of ICH; still obtained results do not deny the possible role of PVS as the marker of stroke.^[28]^ And yet it is important to note that, despite numerous magnetic resonance studies, as well as in vivo and *ex vivo* animal tracer models, the shape of PVS is not defined and appears to adapt to hydraulic resistance of the brain.^[33]^ Moreover, due to the significant artifacts from processing, postmortem histology is also unable to underline to the exact timing when enlargement occurs.^[34]^ Therefore, using PVS as a diagnostic marker might lack specificity, crucial for the timely diagnosis. New experimental studies are needed to overcome above limitations before the planning of clinical research that involve risk to ICH patients.

Strengths of MR approach were fully utilized in this study, with the use of 4 comprehensive methods for the main analysis and 4 specialized methods for sensitivity screening. Newest GWAS data were obtained from the most recent studies, and the PVS in different brain regions were assessed, reflecting the later findings that basal ganglia region is the most common area for ICH.^[3]^ Sensitivity analysis showed that results were not affected by heterogeneity and pleiotropy, with the MR‐PRESSO outliers test specifically designed to screen for outliers, minimizing its effect on causality. Still, the 2-sample approach could simultaneously increase the value of results by excluding confounders, and limit the interpretation possibilities, as PVS function is closely linked to vascular factors.

Some other limitations apply to our study. Firstly, the number of included SNPs was not very big and more data is needed to conclusively debate the role of PVS in ICH. Secondly, in order to adequately compare exposure and outcome, only GWAS data of individuals with European ancestry was included. The association between ICH and PVS in other ethnic groups needs to be further validated, especially as there is some evidence that shape and size of PVS differ with ethnicity,^[18]^ while ICH morbidity has distinct regional differences.^[3]^ Finally, although inclusion criterion for IVs in this study for *F*-statistic was to be over 10, which translates to the adequate validity, overall *F*-statistic values did not exceed 100, which might have led to the weak negative bias, and findings should be interpreted with caution, taking into account all factors discussed above.

## 5. Conclusion

In conclusion, findings of this study do not support the decisive role of PVS enlargement in the development of ICH. Using PVS as a diagnostic marker might lack specificity, crucial for the timely diagnosis, and new experimental studies are needed to overcome this limitation before the planning of excessive diagnostic procedures in the risk populations.

## Author contributions

**Conceptualization:** Wentao Yan, Xiuhua He.

**Data curation:** Wentao Yan, Xiuhua He, Guochao Hu.

**Formal analysis:** Wentao Yan, Guochao Hu.

**Funding acquisition:** Wentao Yan.

**Investigation:** Wentao Yan, Guochao Hu, Kui Chen.

**Methodology:** Wentao Yan, Xiuhua He.

**Supervision:** Xiuhua He, Guanjun Wang.

**Validation:** Wentao Yan, Kui Chen.

**Writing – original draft:** Wentao Yan, Xiuhua He, Guochao Hu, Kui Chen, Guanjun Wang.

**Writing – review & editing:** Wentao Yan, Xiuhua He, Guochao Hu, Kui Chen, Guanjun Wang.

## Supplementary Material



## References

[1] Feigin VL Norrving B Mensah GA. Global burden of stroke. Circ Res. 2017;120:439–48. 28154096 10.1161/CIRCRESAHA.116.308413

[2] Wang S Zou XL Wu LX. Epidemiology of intracerebral hemorrhage: a systematic review and meta-analysis. Front Neurol. 2022;13:915813. 36188383 10.3389/fneur.2022.915813 PMC9523083

[3] GBD 2019 Stroke Collaborators. Global, regional, and national burden of stroke and its risk factors, 1990-2019: a systematic analysis for the Global Burden of Disease Study 2019. Lancet Neurol. 2021;20:795–820. 34487721 10.1016/S1474-4422(21)00252-0 PMC8443449

[4] Sterenstein A Garg R. The impact of sex on epidemiology, management, and outcome of spontaneous intracerebral hemorrhage (sICH). J Stroke Cerebrovasc Dis. 2024;33:107755. 38705497 10.1016/j.jstrokecerebrovasdis.2024.107755

[5] Carhuapoma L Murthy S Shah VA. Outcome trajectories after intracerebral hemorrhage. Semin Neurol. 2024;44:298–307. 38788763 10.1055/s-0044-1787104

[6] Wardlaw JM Benveniste H Nedergaard M; Colleagues from the Fondation Leducq Transatlantic Network of Excellence on the Role of the Perivascular Space in Cerebral Small Vessel Disease. Perivascular spaces in the brain: anatomy, physiology and pathology. Nat Rev Neurol. 2020;16:137–53. 32094487 10.1038/s41582-020-0312-z

[7] van Veluw SJ Perosa V. The perivascular space race: understanding their role in brain clearance. Neurology. 2022;98:95–6. 34810242 10.1212/WNL.0000000000013105

[8] Mestre H Mori Y Nedergaard M. The brain’s glymphatic system: current controversies. Trends Neurosci. 2020;43:458–66. 32423764 10.1016/j.tins.2020.04.003 PMC7331945

[9] Boulouis G Charidimou A Pasi M. Hemorrhage recurrence risk factors in cerebral amyloid angiopathy: comparative analysis of the overall small vessel disease severity score versus individual neuroimaging markers. J Neurol Sci. 2017;380:64–7. 28870591 10.1016/j.jns.2017.07.015 PMC5678990

[10] Gutierrez J Elkind MSV Dong C. Brain perivascular spaces as biomarkers of vascular risk: results from the Northern Manhattan Study. AJNR Am J Neuroradiol. 2017;38:862–7. 28341719 10.3174/ajnr.A5129 PMC5433915

[11] Ference BA Holmes MV Smith GD. Using Mendelian randomization to improve the design of randomized trials. Cold Spring Harb Perspect Med. 2021;11:a040980. 33431510 10.1101/cshperspect.a040980 PMC8247560

[12] Lamina C. Mendelian randomization: principles and its usage in Lp(a) research. Atherosclerosis. 2022;349:36–41. 35606074 10.1016/j.atherosclerosis.2022.04.013

[13] Zou X Wang L Wang S Zhang L. Mendelian randomization study and meta-analysis exploring the causality of age at menarche and the risk of intracerebral hemorrhage and ischemic stroke. CNS Neurosci Ther. 2023;29:3043–52. 37170723 10.1111/cns.14245 PMC10493675

[14] Song Y Zou X Zeng Y Zhang L Mao X. Inflammatory bowel disease and the risk of intracerebral hemorrhage: a Mendelian randomization study and meta-analysis. Immun Inflammation Dis. 2023;11:e1048. 10.1002/iid3.1048 PMC10580698 37904677

[15] Skrivankova VW Richmond RC Woolf BAR. Strengthening the reporting of observational studies in epidemiology using Mendelian randomization: the STROBE-MR statement. JAMA. 2021;326:1614–21. 34698778 10.1001/jama.2021.18236

[16] Qing X Jiang J Yuan C Wang K. Mendelian randomization analysis identifies a genetic casual association between circulating C-reactive protein and intracerebral hemorrhage. J Stroke Cerebrovasc Dis. 2024;33:107554. 38176227 10.1016/j.jstrokecerebrovasdis.2023.107554

[17] Duperron MG Knol MJ Le Grand Q; CHARGE Consortium. Genomics of perivascular space burden unravels early mechanisms of cerebral small vessel disease. Nat Med. 2023;29:950–62. 37069360 10.1038/s41591-023-02268-w PMC10115645

[18] Shih NC Barisano G Lincoln KD Mack WJ Sepehrband F Choupan J. Effects of sleep on brain perivascular space in a cognitively healthy population. Sleep Med. 2023;111:170–9. 37782994 10.1016/j.sleep.2023.09.024 PMC10591884

[19] Burgess S Butterworth A Thompson SG. Mendelian randomization analysis with multiple genetic variants using summarized data. Genet Epidemiol. 2013;37:658–65. 24114802 10.1002/gepi.21758 PMC4377079

[20] Bowden J Del Greco MF Minelli C. Improving the accuracy of two-sample summary-data Mendelian randomization: moving beyond the NOME assumption. Int J Epidemiol. 2019;48:728–42. 30561657 10.1093/ije/dyy258 PMC6659376

[21] Burgess S Thompson SG. Interpreting findings from Mendelian randomization using the MR-Egger method. Eur J Epidemiol. 2017;32:377–89. 28527048 10.1007/s10654-017-0255-x PMC5506233

[22] Bowden J Davey Smith G Haycock PC Burgess S. Consistent estimation in Mendelian randomization with some invalid instruments using a weighted median estimator. Genet Epidemiol. 2016;40:304–14. 27061298 10.1002/gepi.21965 PMC4849733

[23] Nolte IM. Metasubtract: an R-package to analytically produce leave-one-out meta-analysis GWAS summary statistics. Bioinformatics. 2020;36:4521–2. 32696040 10.1093/bioinformatics/btaa570 PMC7750933

[24] Lau KK Li L Lovelock CE. Clinical correlates, ethnic differences, and prognostic implications of perivascular spaces in transient ischemic attack and ischemic stroke. Stroke. 2017;48:1470–7. 28495831 10.1161/STROKEAHA.117.016694 PMC5436733

[25] Ghadirpour R. The aftermath of ischemic stroke: inflammation, comorbidity, and disability. Eur J Neurodegenerative Dis. 2023;12:35–40.

[26] Duperron MG Tzourio C Schilling S. High dilated perivascular space burden: a new MRI marker for risk of intracerebral hemorrhage. Neurobiol Aging. 2019;84:158–65. 31629114 10.1016/j.neurobiolaging.2019.08.031

[27] Yamasaki T Ikawa F Ichihara N. Factors associated with the location of perivascular space enlargement in middle-aged individuals undergoing brain screening in Japan. Clin Neurol Neurosurg. 2022;223:107497. 36356441 10.1016/j.clineuro.2022.107497

[28] Best JG Ambler G Wilson D. Clinical associations and prognostic value of MRI-visible perivascular spaces in patients with ischemic stroke or TIA: a pooled analysis. Neurology. 2024;102:e207795. 38165371 10.1212/WNL.0000000000207795 PMC10834118

[29] Gouveia-Freitas K Bastos-Leite AJ. Perivascular spaces and brain waste clearance systems: relevance for neurodegenerative and cerebrovascular pathology. Neuroradiology. 2021;63:1581–97. 34019111 10.1007/s00234-021-02718-7 PMC8460534

[30] Lysen TS Yilmaz P Dubost F. Sleep and perivascular spaces in the middle-aged and elderly population. J Sleep Res. 2022;31:e13485. 34549850 10.1111/jsr.13485 PMC9285071

[31] Liaunardy-Jopeace A Murton BL Mahesh M Chin JW James JR. Encoding optical control in LCK kinase to quantitatively investigate its activity in live cells. Nat Struct Mol Biol. 2017;24 :1155–63. 29083415 10.1038/nsmb.3492 PMC5736103

[32] Ferrulli T Massi F De Lutiis MA. Physiopathology of tyrosine kinase receptors in the brain. Eur J Neurodegenerative Dis. 2024;13:90–3.

[33] Hablitz LM Vinitsky HS Sun Q. Increased glymphatic influx is correlated with high EEG delta power and low heart rate in mice under anesthesia. Sci Adv. 2019;5:eaav5447. 30820460 10.1126/sciadv.aav5447 PMC6392807

[34] Albargothy NJ Johnston DA MacGregor-Sharp M. Convective influx/glymphatic system: tracers injected into the CSF enter and leave the brain along separate periarterial basement membrane pathways. Acta Neuropathol. 2018;136:139–52. 29754206 10.1007/s00401-018-1862-7 PMC6015107

