# Supplementary material for: The causal relationship between enlarged perivascular spaces and intracerebral hemorrhage: A 2-sample Mendelian randomization study
Source: Medicine (Baltimore). 2025 Jun 6;104(23):e42658. doi: 10.1097/MD.0000000000042658 (PMC12150965; doi:10.1097/MD.0000000000042658)
Supplement: Supplementary file 2 [file medi-104-e42658-s002.docx]

**Supplemental Digital Content Table 2**. List of Single Nucleotide Polymorphisms (SNPs) included as Instrumental Variables (IVs).

| **Exposure** | **N.SNPs** | **Included SNPs** | **Excluded SNPs** | **F-statistic mean (min, max)** |
| --- | --- | --- | --- | --- |
| Extensive hippocampal perivascular space burden | 12 | rs2022392, rs6540873, rs7596872, rs111833237, rs73062730, rs79850821, rs7375471, rs238449, rs6879760, rs9345054, rs7076119, rs11500477, rs2284356 | rs11500477, rs2022392 | 23.93 (14.66, 49.38) |
| Extensive basal ganglia perivascular space burden | 13 | rs10494988, rs7596872, rs13079464, rs4678162, rs6444747, rs4959689, rs10954468, rs62509329, rs10817108, rs12417836, rs8041189, rs1126642, rs2425884, rs6011998 |  | 38.94 (21.78, 90.78) |
| Extensive white matter perivascular space burden | 14 | rs10494988, rs7596872, rs13079464, rs4678162, rs6444747, rs4959689, rs10954468, rs62509329, rs10817108, rs12417836, rs8041189, rs1126642, rs2425884, rs6011998 | rs13079464 | 38.94 (21.78, 90.78) |

This table lists the single nucleotide polymorphisms (SNPs) included as instrumental variables for each exposure type—extensive hippocampal, basal ganglia, and white matter perivascular space burdens. It specifies the number of SNPs used (N.SNPs), the included SNPs, excluded SNPs, and the mean F-statistic along with its minimum and maximum values. The inclusion of these SNPs is crucial for assessing the strength of the instruments in Mendelian randomization studies.
